# Desoxyhemigossypol 6-methyl ether

**DOI:** 10.1107/S1600536813002304

**Published:** 2013-01-31

**Authors:** Vyacheslav V. Uzbekov, Samat A. Talipov, Bakhtiyar T. Ibragimov, Robert D. Stipanovic, Jinggao Liu

**Affiliations:** a Institute of Bioorganic Chemistry, Academy of Sciences of Uzbekistan, M. Ulugbek str. 83, Tashkent, 100125 Uzbekistan; b Southern Plains Agricultural Research Center, Agricultural Research, Service, USDA, College Station, TX 77845, USA

## Abstract

The title sesquiterpene [systematic name: 6-methoxy-10-methyl-7-(propan-2-yl)-2-oxatricyclo[6.3.1.0^4,12^]dodeca-1(11),4,6,8(12),9-pentaen-5-ol], C_16_H_18_O_3_, was isolated from pathogen-infected stele tissue of *Gossypium barbadense*. There are two mol­ecules in the asymmetric unit and the dihedral angle between their naphtho­furan systems is 86.48 (2)°. In the crystal, O—H⋯O hydrogen bonds between the hy­droxy groups and etheric O atoms link the mol­ecules into centrosymmetric tetra­mers. These tetra­mers are assembled into (010) layers *via* stacking inter­actions between the naphtho­furan systems [inter­planar distance 3.473 (3) Å] and short C—H⋯O contacts.

## Related literature

For the isolation and chemical structure determination of related cotton sesquiterpenoid phytoalexins and their inter­mediates, see: Bell *et al.* (1975 ▶); Stipanovic *et al.* (1975 ▶). For the role of terpenoid aldehydes as phytoalexins (active defense agents) in response to infection by wilt fungi, see: Mace (1978 ▶). For information on the mechanism of action, see: Mace *et al.* (1995 ▶). For the mechanism of *O*-methyl­ation of desoxy­hemigossypol, see: Liu *et al.* (2002 ▶). For general information about anti­microbial compounds produced by cotton, see: Bell (1995 ▶).
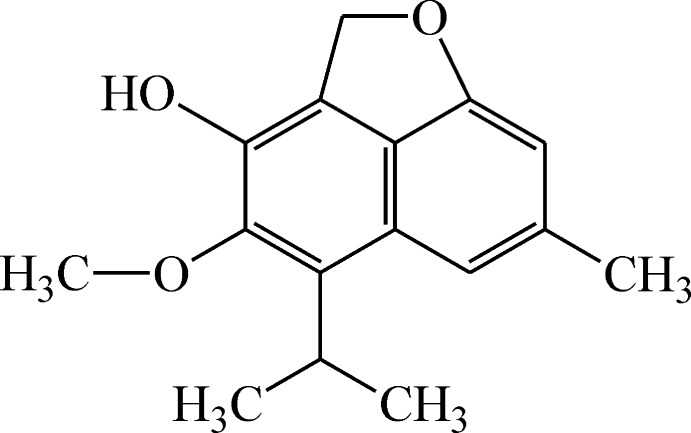


## Experimental

### 

#### Crystal data

C_16_H_18_O_3_
*M* *_r_* = 258.30
Triclinic, 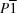

*a* = 10.0275 (5) Å
*b* = 11.1058 (6) Å
*c* = 13.2938 (8) Å
α = 107.797 (5)°
β = 103.896 (5)°
γ = 90.435 (4)°
*V* = 1363.08 (13) Å^3^
*Z* = 4
Cu *K*α radiation
μ = 0.69 mm^−1^
*T* = 293 K
0.40 × 0.34 × 0.28 mm

#### Data collection

Oxford Diffraction Xcalibur Ruby diffractometer
Absorption correction: multi-scan (*CrysAlis PRO*; Oxford Diffraction, 2009 ▶) *T*_min_ = 0.925, *T*_max_ = 1.000
10119 measured reflections
5476 independent reflections
3775 reflections with *I* > 2σ(*I*)
*R*_int_ = 0.027

#### Refinement

*R*[*F* ^2^ > 2σ(*F* ^2^)] = 0.050
*wR*(*F* ^2^) = 0.158
*S* = 1.04
5476 reflections
354 parameters
H-atom parameters constrained
Δρ_max_ = 0.45 e Å^−3^
Δρ_min_ = −0.22 e Å^−3^

### 

Data collection: *CrysAlis PRO* (Oxford Diffraction, 2009 ▶); cell refinement: *CrysAlis PRO*; data reduction: *CrysAlis PRO*; program(s) used to solve structure: *SHELXS97* (Sheldrick, 2008 ▶); program(s) used to refine structure: *SHELXL97* (Sheldrick, 2008 ▶); molecular graphics: *XP* in *SHELXTL* (Sheldrick, 2008 ▶); software used to prepare material for publication: *SHELXL97*.

## Supplementary Material

Crystal structure: contains datablock(s) I, global. DOI: 10.1107/S1600536813002304/gk2530sup1.cif

Structure factors: contains datablock(s) I. DOI: 10.1107/S1600536813002304/gk2530Isup2.hkl

Supplementary material file. DOI: 10.1107/S1600536813002304/gk2530Isup3.cml

Additional supplementary materials:  crystallographic information; 3D view; checkCIF report

## Figures and Tables

**Table 1:** Hydrogen-bond geometry (Å, °)

*D*—H⋯*A*	*D*—H	H⋯*A*	*D*⋯*A*	*D*—H⋯*A*
O2—H2*A*⋯O3	0.82	2.28	2.723 (2)	114
O2—H2*A*⋯O4^i^	0.82	2.11	2.809 (2)	144
O5—H5⋯O3	0.82	2.13	2.7843 (19)	137
O5—H5⋯O6	0.82	2.27	2.719 (2)	115
C12—H12*B*⋯O2^ii^	0.97	2.48	3.445 (3)	171

Symmetry codes: (i) 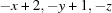; (ii) 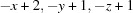.

**Fig. 1.**
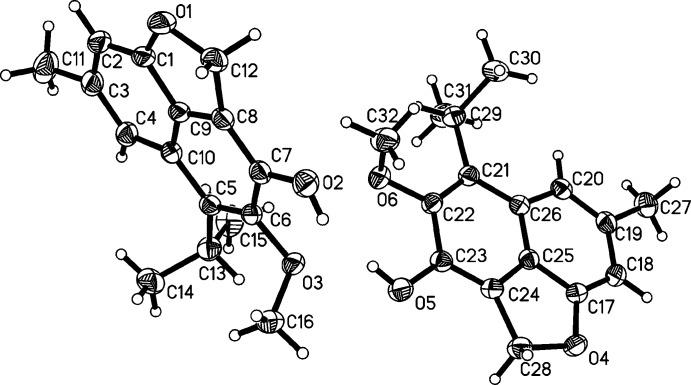
The molecular structure of title compound with the atom-numbering scheme. Displacement ellipsoids are drawn at the 50% probability level.

**Fig. 2.**
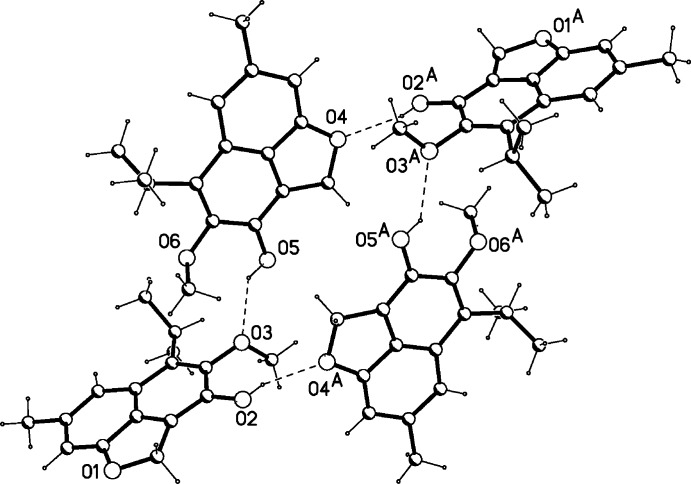
Hydrogen bonded tetramer of the title compound. Atoms with the A label are generated by the symmetry operation 2-x, 1-y, -z.

**Fig. 3.**
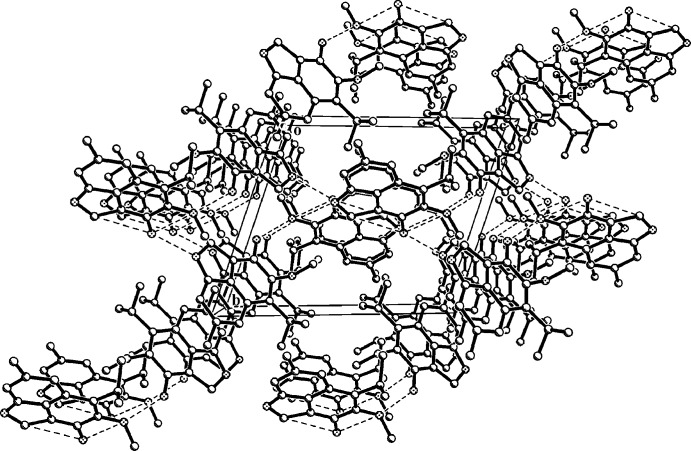
A packing diagram for the title compound. Short contacts are represented by dashed lines.

## supplementary crystallographic information

### Comment

The title sesquiterpene compound with systematic name 3-hydroxy-5-(1-methylethyl)-4-methoxy-7-methyl-*2H*-naphtho[*1,8-bc*]furan, was isolated from pathogen-infected stele tissue of *Gossypium barbadense* (fine-fibre Egyptian cotton) where it plays a role as a phytoalexin (active defense agent in response to infection by wilt fungi). The plant is widely cultivated in cotton-producing countries such as USA, Mexico, China, Uzbekistan and Egypt. Here we report its crystal structure.

The numbering scheme of atoms is shown in Fig. 1. The 12 non-hydrogen atoms of the naphthofuran system in each of the two symmetry independent molecules are virtually coplanar with r.m.s. values of 0.020 and 0.028 Å. Hydrogen bonds between hydroxy group and etheric oxygen atom link the molecules into centrosymmetric tertramers (Table 1, Fig. 2). These tertramers are assembled into (010) layers *via* stacking interactions between the naphthofuran systems [interplanar distance 3.473 (3) Å] and short C—H···O interactions.

### Experimental

The title compound was extracted from *V. dahliae* infected cotton stems and the crude extract, was purified by column and TLC chromatography as described by Bell *et al.*(1975), and crystalized from CHCl_3_ and hexane to give crystals (m.p. 429-433 K). These crystals were further purified by semi-preparative reverse phase HPLC (Agilent 1100 HPLC system; Zorbax Eclipse XDB C8 column; Agilent Technologies Inc., USA) with dimensions 9.4 × 250 mm and particle size 5 µm. The column was eluted using a linear gradient of H_2_O(A)/CH_3_CN(B) [LC grade, Sigma-Aldrich, DE], both with 0.01% *ortho*-phosphoric acid pH 2.5 (from 55 to 95% B for 20 minutes) at a flow rate of 4 ml/min. Crystals suitable for X-ray diffraction analysis were obtained by slow evaporation of the HPLC eluent.

### Refinement

All H atoms were included in calculated positions, with C—H bond distances of 0.98 Å (CH), 0.97 Å (CH_2_), 0.96 Å (CH_3_), 0.93 Å (aromatic) and O—H = 0.82 Å and refined in a riding model approximation with *U*_iso_(H) = 1.5*U*_eq_(C_methyl_,O) and *U*_iso_(H) = 1.2*U*_eq_(C) for the remaining H atoms.

### Crystal data

**Table:**

C_16_H_18_O_3_	*Z* = 4
*M**_r_* = 258.30	*F*(000) = 552
Triclinic, *P*1	*D*_x_ = 1.259 Mg m^−^^3^
Hall symbol: -P 1	Cu *K*α radiation, λ = 1.54184 Å
*a* = 10.0275 (5) Å	Cell parameters from 3316 reflections
*b* = 11.1058 (6) Å	θ = 3.6–75.7°
*c* = 13.2938 (8) Å	µ = 0.69 mm^−^^1^
α = 107.797 (5)°	*T* = 293 K
β = 103.896 (5)°	Block, colourless
γ = 90.435 (4)°	0.40 × 0.34 × 0.28 mm
*V* = 1363.08 (13) Å^3^	

### Data collection

**Table:**

Oxford Diffraction Xcalibur Ruby diffractometer	5476 independent reflections
Radiation source: fine-focus sealed tube	3775 reflections with *I* > 2σ(*I*)
Graphite monochromator	*R*_int_ = 0.027
Detector resolution: 10.2576 pixels mm^-1^	θ_max_ = 75.9°, θ_min_ = 3.6°
ω scans	*h* = −11→12
Absorption correction: multi-scan (*CrysAlis PRO*; Oxford Diffraction, 2009)	*k* = −13→12
*T*_min_ = 0.925, *T*_max_ = 1.000	*l* = −16→16
10119 measured reflections	

### Refinement

**Table:**

Refinement on *F*^2^	Secondary atom site location: difference Fourier map
Least-squares matrix: full	Hydrogen site location: inferred from neighbouring sites
*R*[*F*^2^ > 2σ(*F*^2^)] = 0.050	H-atom parameters constrained
*wR*(*F*^2^) = 0.158	*w* = 1/[σ^2^(*F*_o_^2^) + (0.0881*P*)^2^ + 0.0965*P*] where *P* = (*F*_o_^2^ + 2*F*_c_^2^)/3
*S* = 1.04	(Δ/σ)_max_ < 0.001
5476 reflections	Δρ_max_ = 0.45 e Å^−^^3^
354 parameters	Δρ_min_ = −0.22 e Å^−^^3^
0 restraints	Extinction correction: *SHELXL*, Fc^*^=kFc[1+0.001xFc^2^λ^3^/sin(2θ)]^-1/4^
Primary atom site location: structure-invariant direct methods	Extinction coefficient: 0.0038 (6)

### Special details

**Table:**

Geometry. All esds (except the esd in the dihedral angle between two l.s. planes) are estimated using the full covariance matrix. The cell esds are taken into account individually in the estimation of esds in distances, angles and torsion angles; correlations between esds in cell parameters are only used when they are defined by crystal symmetry. An approximate (isotropic) treatment of cell esds is used for estimating esds involving l.s. planes.
Refinement. Refinement of *F*^2^ against ALL reflections. The weighted *R*-factor *wR* and goodness of fit *S* are based on *F*^2^, conventional *R*-factors *R* are based on *F*, with *F* set to zero for negative *F*^2^. The threshold expression of *F*^2^ > σ(*F*^2^) is used only for calculating *R*-factors(gt) *etc*. and is not relevant to the choice of reflections for refinement. *R*-factors based on *F*^2^ are statistically about twice as large as those based on *F*, and *R*- factors based on ALL data will be even larger.

### Fractional atomic coordinates and isotropic or equivalent isotropic displacement parameters (Å^2^)

**Table:**

	*x*	*y*	*z*	*U*_iso_*/*U*_eq_	
C1	0.6372 (2)	0.6300 (2)	0.60266 (16)	0.0584 (5)	
C2	0.5457 (3)	0.7031 (2)	0.64751 (18)	0.0701 (7)	
H2	0.5440	0.7154	0.7196	0.084*	
C3	0.4517 (3)	0.7608 (2)	0.5804 (2)	0.0673 (6)	
C4	0.4535 (2)	0.7425 (2)	0.47364 (18)	0.0583 (5)	
H4	0.3927	0.7831	0.4328	0.070*	
C5	0.56004 (19)	0.62981 (17)	0.31437 (15)	0.0461 (4)	
C6	0.65818 (18)	0.54699 (17)	0.28745 (14)	0.0448 (4)	
C7	0.74826 (19)	0.49542 (18)	0.36144 (16)	0.0479 (4)	
C8	0.73732 (19)	0.52956 (19)	0.46605 (16)	0.0492 (4)	
C9	0.63691 (19)	0.61040 (18)	0.49337 (15)	0.0487 (4)	
C10	0.54617 (19)	0.66320 (18)	0.42417 (15)	0.0486 (4)	
C11	0.3488 (3)	0.8409 (3)	0.6302 (3)	0.0941 (9)	
H11A	0.3084	0.8905	0.5848	0.141*	
H11B	0.3947	0.8965	0.7016	0.141*	
H11C	0.2777	0.7868	0.6359	0.141*	
C12	0.8076 (2)	0.4986 (2)	0.56747 (18)	0.0641 (6)	
H12A	0.7977	0.4079	0.5551	0.077*	
H12B	0.9050	0.5272	0.5900	0.077*	
C13	0.4747 (2)	0.6820 (2)	0.22867 (18)	0.0594 (5)	
H13	0.5046	0.6427	0.1619	0.071*	
C14	0.3207 (2)	0.6418 (3)	0.1997 (2)	0.0762 (7)	
H14A	0.3072	0.5517	0.1843	0.114*	
H14B	0.2747	0.6640	0.1365	0.114*	
H14C	0.2835	0.6845	0.2600	0.114*	
C15	0.5064 (3)	0.8232 (2)	0.2559 (3)	0.0853 (8)	
H15A	0.4851	0.8668	0.3238	0.128*	
H15B	0.4517	0.8515	0.1989	0.128*	
H15C	0.6024	0.8411	0.2623	0.128*	
C16	0.5963 (2)	0.4046 (2)	0.10614 (18)	0.0665 (6)	
H16A	0.6233	0.3830	0.0387	0.100*	
H16B	0.5017	0.4238	0.0935	0.100*	
H16C	0.6065	0.3342	0.1342	0.100*	
O1	0.73758 (17)	0.56665 (17)	0.64974 (12)	0.0721 (5)	
O2	0.84360 (15)	0.41512 (15)	0.33045 (13)	0.0626 (4)	
H2A	0.8335	0.3970	0.2643	0.094*	
O3	0.68208 (14)	0.51333 (13)	0.18394 (10)	0.0530 (3)	
C17	1.1325 (2)	0.81302 (19)	−0.05314 (15)	0.0498 (4)	
C18	1.2223 (2)	0.9049 (2)	−0.05337 (17)	0.0561 (5)	
H18	1.2599	0.8966	−0.1124	0.067*	
C19	1.2561 (2)	1.0143 (2)	0.04066 (18)	0.0555 (5)	
C20	1.2035 (2)	1.02680 (19)	0.12981 (17)	0.0530 (5)	
H20	1.2300	1.0994	0.1903	0.064*	
C21	1.04433 (19)	0.92410 (18)	0.21481 (16)	0.0480 (4)	
C22	0.9539 (2)	0.81841 (18)	0.19220 (15)	0.0485 (4)	
C23	0.9187 (2)	0.71749 (18)	0.09065 (15)	0.0490 (4)	
C24	0.9836 (2)	0.72145 (17)	0.01286 (15)	0.0480 (4)	
C25	1.07617 (18)	0.82592 (17)	0.03528 (15)	0.0452 (4)	
C26	1.10907 (18)	0.93059 (17)	0.13117 (15)	0.0456 (4)	
C27	1.3522 (3)	1.1198 (2)	0.0407 (2)	0.0745 (7)	
H27A	1.3271	1.2002	0.0809	0.112*	
H27B	1.3452	1.1177	−0.0332	0.112*	
H27C	1.4453	1.1088	0.0743	0.112*	
C28	0.9801 (2)	0.6354 (2)	−0.09991 (17)	0.0599 (5)	
H28A	1.0057	0.5518	−0.0983	0.072*	
H28B	0.8891	0.6269	−0.1488	0.072*	
C29	1.0730 (2)	1.0259 (2)	0.32693 (17)	0.0585 (5)	
H29	1.0156	0.9971	0.3667	0.070*	
C30	1.2201 (2)	1.0329 (2)	0.39328 (19)	0.0680 (6)	
H30A	1.2413	0.9502	0.3977	0.102*	
H30B	1.2305	1.0910	0.4655	0.102*	
H30C	1.2818	1.0619	0.3587	0.102*	
C31	1.0288 (3)	1.1533 (2)	0.3244 (2)	0.0766 (7)	
H31A	1.0832	1.1876	0.2876	0.115*	
H31B	1.0417	1.2091	0.3978	0.115*	
H31C	0.9332	1.1450	0.2863	0.115*	
C32	0.9621 (3)	0.7427 (3)	0.3406 (2)	0.0781 (7)	
H32A	0.9789	0.6596	0.2989	0.117*	
H32B	0.9104	0.7354	0.3908	0.117*	
H32C	1.0485	0.7914	0.3804	0.117*	
O4	1.08204 (16)	0.69916 (14)	−0.13428 (11)	0.0607 (4)	
O5	0.82418 (17)	0.61951 (15)	0.07111 (12)	0.0663 (4)	
H5	0.7976	0.6270	0.1263	0.099*	
O6	0.88542 (15)	0.80485 (15)	0.26829 (12)	0.0612 (4)	

### Atomic displacement parameters (Å^2^)

**Table:**

	*U*^11^	*U*^22^	*U*^33^	*U*^12^	*U*^13^	*U*^23^
C1	0.0624 (13)	0.0646 (13)	0.0392 (10)	−0.0247 (10)	0.0058 (9)	0.0100 (9)
C2	0.0794 (16)	0.0761 (15)	0.0450 (11)	−0.0301 (12)	0.0200 (11)	0.0029 (10)
C3	0.0718 (14)	0.0577 (13)	0.0658 (14)	−0.0165 (10)	0.0293 (12)	0.0015 (11)
C4	0.0589 (12)	0.0535 (11)	0.0606 (12)	−0.0032 (9)	0.0210 (10)	0.0111 (10)
C5	0.0459 (10)	0.0477 (10)	0.0435 (10)	−0.0038 (8)	0.0066 (8)	0.0166 (8)
C6	0.0453 (10)	0.0487 (10)	0.0402 (9)	−0.0074 (8)	0.0095 (7)	0.0150 (8)
C7	0.0433 (10)	0.0492 (10)	0.0505 (11)	−0.0040 (8)	0.0096 (8)	0.0168 (8)
C8	0.0435 (9)	0.0562 (11)	0.0468 (10)	−0.0083 (8)	0.0035 (8)	0.0213 (8)
C9	0.0492 (10)	0.0532 (11)	0.0391 (9)	−0.0138 (8)	0.0065 (8)	0.0123 (8)
C10	0.0478 (10)	0.0487 (10)	0.0468 (10)	−0.0070 (8)	0.0111 (8)	0.0125 (8)
C11	0.112 (2)	0.0758 (18)	0.097 (2)	−0.0006 (15)	0.0625 (18)	0.0033 (15)
C12	0.0561 (12)	0.0803 (15)	0.0551 (12)	−0.0094 (11)	−0.0004 (10)	0.0319 (11)
C13	0.0608 (12)	0.0671 (13)	0.0563 (12)	0.0090 (10)	0.0128 (10)	0.0295 (10)
C14	0.0596 (13)	0.0940 (18)	0.0718 (15)	0.0061 (12)	−0.0010 (11)	0.0353 (14)
C15	0.0937 (19)	0.0731 (17)	0.108 (2)	0.0165 (14)	0.0338 (17)	0.0493 (16)
C16	0.0740 (14)	0.0662 (14)	0.0494 (12)	−0.0038 (11)	0.0119 (10)	0.0072 (10)
O1	0.0736 (10)	0.0946 (12)	0.0453 (8)	−0.0151 (9)	0.0010 (7)	0.0295 (8)
O2	0.0562 (8)	0.0723 (10)	0.0627 (9)	0.0146 (7)	0.0165 (7)	0.0249 (8)
O3	0.0590 (8)	0.0579 (8)	0.0434 (7)	−0.0053 (6)	0.0169 (6)	0.0151 (6)
C17	0.0510 (10)	0.0525 (11)	0.0445 (10)	0.0036 (8)	0.0108 (8)	0.0145 (8)
C18	0.0536 (11)	0.0669 (13)	0.0525 (11)	0.0010 (9)	0.0184 (9)	0.0220 (10)
C19	0.0473 (10)	0.0590 (12)	0.0614 (12)	−0.0027 (9)	0.0104 (9)	0.0234 (10)
C20	0.0490 (10)	0.0488 (10)	0.0564 (12)	−0.0015 (8)	0.0088 (9)	0.0134 (9)
C21	0.0468 (10)	0.0492 (10)	0.0462 (10)	0.0066 (8)	0.0114 (8)	0.0130 (8)
C22	0.0501 (10)	0.0530 (11)	0.0443 (10)	0.0060 (8)	0.0139 (8)	0.0166 (8)
C23	0.0517 (10)	0.0465 (10)	0.0475 (10)	−0.0015 (8)	0.0087 (8)	0.0162 (8)
C24	0.0526 (10)	0.0457 (10)	0.0433 (10)	0.0005 (8)	0.0076 (8)	0.0140 (8)
C25	0.0427 (9)	0.0488 (10)	0.0457 (10)	0.0035 (7)	0.0091 (8)	0.0189 (8)
C26	0.0451 (9)	0.0444 (9)	0.0467 (10)	0.0045 (7)	0.0094 (8)	0.0151 (8)
C27	0.0697 (15)	0.0747 (16)	0.0810 (16)	−0.0162 (12)	0.0205 (13)	0.0268 (13)
C28	0.0707 (13)	0.0555 (12)	0.0496 (11)	−0.0083 (10)	0.0152 (10)	0.0116 (9)
C29	0.0632 (13)	0.0551 (12)	0.0500 (11)	0.0036 (9)	0.0145 (10)	0.0065 (9)
C30	0.0680 (14)	0.0671 (14)	0.0559 (13)	0.0026 (11)	0.0024 (11)	0.0113 (10)
C31	0.0866 (17)	0.0659 (15)	0.0707 (15)	0.0164 (12)	0.0193 (13)	0.0126 (12)
C32	0.0918 (18)	0.0867 (17)	0.0575 (13)	−0.0204 (14)	0.0101 (12)	0.0328 (13)
O4	0.0726 (9)	0.0588 (8)	0.0475 (8)	−0.0071 (7)	0.0220 (7)	0.0076 (6)
O5	0.0794 (10)	0.0641 (9)	0.0538 (8)	−0.0213 (8)	0.0213 (8)	0.0138 (7)
O6	0.0636 (9)	0.0718 (9)	0.0521 (8)	−0.0019 (7)	0.0222 (7)	0.0197 (7)

### Geometric parameters (Å, º)

**Table:**

C1—C2	1.354 (3)	C17—C18	1.357 (3)
C1—O1	1.370 (3)	C17—O4	1.383 (2)
C1—C9	1.401 (3)	C17—C25	1.392 (3)
C2—C3	1.432 (4)	C18—C19	1.420 (3)
C2—H2	0.9300	C18—H18	0.9300
C3—C4	1.376 (3)	C19—C20	1.380 (3)
C3—C11	1.510 (3)	C19—C27	1.511 (3)
C4—C10	1.422 (3)	C20—C26	1.429 (3)
C4—H4	0.9300	C20—H20	0.9300
C5—C6	1.387 (3)	C21—C22	1.388 (3)
C5—C10	1.433 (3)	C21—C26	1.435 (3)
C5—C13	1.520 (3)	C21—C29	1.530 (3)
C6—O3	1.391 (2)	C22—O6	1.394 (2)
C6—C7	1.427 (3)	C22—C23	1.429 (3)
C7—C8	1.357 (3)	C23—O5	1.358 (2)
C7—O2	1.363 (2)	C23—C24	1.359 (3)
C8—C9	1.393 (3)	C24—C25	1.391 (3)
C8—C12	1.504 (3)	C24—C28	1.504 (3)
C9—C10	1.398 (3)	C25—C26	1.404 (3)
C11—H11A	0.9600	C27—H27A	0.9600
C11—H11B	0.9600	C27—H27B	0.9600
C11—H11C	0.9600	C27—H27C	0.9600
C12—O1	1.460 (3)	C28—O4	1.472 (2)
C12—H12A	0.9700	C28—H28A	0.9700
C12—H12B	0.9700	C28—H28B	0.9700
C13—C15	1.510 (3)	C29—C31	1.494 (3)
C13—C14	1.526 (3)	C29—C30	1.514 (3)
C13—H13	0.9800	C29—H29	0.9800
C14—H14A	0.9600	C30—H30A	0.9600
C14—H14B	0.9600	C30—H30B	0.9600
C14—H14C	0.9600	C30—H30C	0.9600
C15—H15A	0.9600	C31—H31A	0.9600
C15—H15B	0.9600	C31—H31B	0.9600
C15—H15C	0.9600	C31—H31C	0.9600
C16—O3	1.436 (2)	C32—O6	1.432 (3)
C16—H16A	0.9600	C32—H32A	0.9600
C16—H16B	0.9600	C32—H32B	0.9600
C16—H16C	0.9600	C32—H32C	0.9600
O2—H2A	0.8200	O5—H5	0.8200
			
C2—C1—O1	128.9 (2)	C18—C17—O4	128.27 (18)
C2—C1—C9	120.4 (2)	C18—C17—C25	121.37 (19)
O1—C1—C9	110.6 (2)	O4—C17—C25	110.36 (17)
C1—C2—C3	117.9 (2)	C17—C18—C19	116.92 (19)
C1—C2—H2	121.1	C17—C18—H18	121.5
C3—C2—H2	121.1	C19—C18—H18	121.5
C4—C3—C2	121.3 (2)	C20—C19—C18	122.20 (19)
C4—C3—C11	120.9 (3)	C20—C19—C27	119.9 (2)
C2—C3—C11	117.8 (2)	C18—C19—C27	117.9 (2)
C3—C4—C10	121.6 (2)	C19—C20—C26	121.41 (19)
C3—C4—H4	119.2	C19—C20—H20	119.3
C10—C4—H4	119.2	C26—C20—H20	119.3
C6—C5—C10	117.24 (17)	C22—C21—C26	117.52 (17)
C6—C5—C13	119.53 (17)	C22—C21—C29	119.33 (18)
C10—C5—C13	123.23 (18)	C26—C21—C29	123.13 (18)
C5—C6—O3	121.02 (16)	C21—C22—O6	121.39 (17)
C5—C6—C7	124.92 (17)	C21—C22—C23	124.49 (18)
O3—C6—C7	113.94 (16)	O6—C22—C23	114.09 (17)
C8—C7—O2	119.94 (18)	O5—C23—C24	120.13 (17)
C8—C7—C6	117.77 (18)	O5—C23—C22	121.93 (18)
O2—C7—C6	122.29 (17)	C24—C23—C22	117.94 (17)
C7—C8—C9	117.79 (18)	C23—C24—C25	118.00 (17)
C7—C8—C12	135.5 (2)	C23—C24—C28	134.59 (18)
C9—C8—C12	106.72 (18)	C25—C24—C28	107.41 (17)
C8—C9—C10	126.68 (18)	C24—C25—C17	109.77 (17)
C8—C9—C1	109.45 (19)	C24—C25—C26	126.36 (18)
C10—C9—C1	123.9 (2)	C17—C25—C26	123.87 (18)
C9—C10—C4	114.85 (18)	C25—C26—C20	114.18 (17)
C9—C10—C5	115.56 (18)	C25—C26—C21	115.56 (17)
C4—C10—C5	129.58 (19)	C20—C26—C21	130.26 (18)
C3—C11—H11A	109.5	C19—C27—H27A	109.5
C3—C11—H11B	109.5	C19—C27—H27B	109.5
H11A—C11—H11B	109.5	H27A—C27—H27B	109.5
C3—C11—H11C	109.5	C19—C27—H27C	109.5
H11A—C11—H11C	109.5	H27A—C27—H27C	109.5
H11B—C11—H11C	109.5	H27B—C27—H27C	109.5
O1—C12—C8	104.78 (18)	O4—C28—C24	103.99 (15)
O1—C12—H12A	110.8	O4—C28—H28A	111.0
C8—C12—H12A	110.8	C24—C28—H28A	111.0
O1—C12—H12B	110.8	O4—C28—H28B	111.0
C8—C12—H12B	110.8	C24—C28—H28B	111.0
H12A—C12—H12B	108.9	H28A—C28—H28B	109.0
C15—C13—C5	112.45 (19)	C31—C29—C30	112.4 (2)
C15—C13—C14	113.3 (2)	C31—C29—C21	114.58 (19)
C5—C13—C14	113.28 (18)	C30—C29—C21	112.46 (17)
C15—C13—H13	105.6	C31—C29—H29	105.5
C5—C13—H13	105.6	C30—C29—H29	105.5
C14—C13—H13	105.6	C21—C29—H29	105.5
C13—C14—H14A	109.5	C29—C30—H30A	109.5
C13—C14—H14B	109.5	C29—C30—H30B	109.5
H14A—C14—H14B	109.5	H30A—C30—H30B	109.5
C13—C14—H14C	109.5	C29—C30—H30C	109.5
H14A—C14—H14C	109.5	H30A—C30—H30C	109.5
H14B—C14—H14C	109.5	H30B—C30—H30C	109.5
C13—C15—H15A	109.5	C29—C31—H31A	109.5
C13—C15—H15B	109.5	C29—C31—H31B	109.5
H15A—C15—H15B	109.5	H31A—C31—H31B	109.5
C13—C15—H15C	109.5	C29—C31—H31C	109.5
H15A—C15—H15C	109.5	H31A—C31—H31C	109.5
H15B—C15—H15C	109.5	H31B—C31—H31C	109.5
O3—C16—H16A	109.5	O6—C32—H32A	109.5
O3—C16—H16B	109.5	O6—C32—H32B	109.5
H16A—C16—H16B	109.5	H32A—C32—H32B	109.5
O3—C16—H16C	109.5	O6—C32—H32C	109.5
H16A—C16—H16C	109.5	H32A—C32—H32C	109.5
H16B—C16—H16C	109.5	H32B—C32—H32C	109.5
C1—O1—C12	108.40 (16)	C17—O4—C28	108.37 (15)
C7—O2—H2A	109.5	C23—O5—H5	109.5
C6—O3—C16	114.15 (14)	C22—O6—C32	112.83 (17)

### Hydrogen-bond geometry (Å, º)

**Table:**

*D*—H···*A*	*D*—H	H···*A*	*D*···*A*	*D*—H···*A*
O2—H2*A*···O3	0.82	2.28	2.723 (2)	114
O2—H2*A*···O4^i^	0.82	2.11	2.809 (2)	144
O5—H5···O3	0.82	2.13	2.7843 (19)	137
O5—H5···O6	0.82	2.27	2.719 (2)	115
C12—H12*B*···O2^ii^	0.97	2.48	3.445 (3)	171

Symmetry codes: (i) −*x*+2, −*y*+1, −*z*; (ii) −*x*+2, −*y*+1, −*z*+1.
